# Transcriptome Tomography for Brain Analysis in the Web-Accessible Anatomical Space

**DOI:** 10.1371/journal.pone.0045373

**Published:** 2012-09-19

**Authors:** Yuko Okamura-Oho, Kazuro Shimokawa, Satoko Takemoto, Asami Hirakiyama, Sakiko Nakamura, Yuki Tsujimura, Masaomi Nishimura, Takeya Kasukawa, Koh-hei Masumoto, Itoshi Nikaido, Yasufumi Shigeyoshi, Hiroki R. Ueda, Gang Song, James Gee, Ryutaro Himeno, Hideo Yokota

**Affiliations:** 1 Advanced Computational Sciences Department, Advanced Science Institute (ASI), RIKEN, Saitama, Japan; 2 Bio-research Infrastructure Construction Team, Advanced Science Institute (ASI), RIKEN, Saitama, Japan; 3 Laboratory for Genome Exploration Research Group, Genomic Sciences Center (GSC), RIKEN Yokohama Institute, Yokohama, Japan; 4 University Center for Information Medicine, Tokyo Medical Dental University, Tokyo, Japan; 5 Functional Genomics Unit, RIKEN Center for Developmental Biology, Kobe, Japan; 6 Laboratory for Systems Biology, RIKEN Center for Developmental Biology, Kobe, Japan; 7 Department of Anatomy and Neurobiology, Kinki University School of Medicine, Osaka, Japan; 8 Penn Image Computing and Science Laboratory, Department of Radiology, University of Pennsylvania, Philadelphia, Pennsylvania, United States of America; Wake Forest School of Medicine, United States of America

## Abstract

Increased information on the encoded mammalian genome is expected to facilitate an integrated understanding of complex anatomical structure and function based on the knowledge of gene products. Determination of gene expression-anatomy associations is crucial for this understanding. To elicit the association in the three-dimensional (3D) space, we introduce a novel technique for comprehensive mapping of endogenous gene expression into a web-accessible standard space: Transcriptome Tomography. The technique is based on conjugation of sequential tissue-block sectioning, all fractions of which are used for molecular measurements of gene expression densities, and the block- face imaging, which are used for 3D reconstruction of the fractions. To generate a 3D map, tissues are serially sectioned in each of three orthogonal planes and the expression density data are mapped using a tomographic technique. This rapid and unbiased mapping technique using a relatively small number of original data points allows researchers to create their own expression maps in the broad anatomical context of the space. In the first instance we generated a dataset of 36,000 maps, reconstructed from data of 61 fractions measured with microarray, covering the whole mouse brain (ViBrism: http://vibrism.riken.jp/3dviewer/ex/index.html) in one month. After computational estimation of the mapping accuracy we validated the dataset against existing data with respect to the expression location and density. To demonstrate the relevance of the framework, we showed disease related expression of Huntington’s disease gene and *Bdnf*. Our tomographic approach is applicable to analysis of any biological molecules derived from frozen tissues, organs and whole embryos, and the maps are spatially isotropic and well suited to the analysis in the standard space (e.g. Waxholm Space for brain-atlas databases). This will facilitate research creating and using open-standards for a molecular-based understanding of complex structures; and will contribute to new insights into a broad range of biological and medical questions.

## Introduction

Increased information on the encoded mammalian genome has led to an integrated understanding of complex biological structure and function based on the knowledge of gene expression [1], [2]. For investigations of the whole tissues or organs such as the mammalian brain, where there are an estimated 25,000 genes expressed [3], combined analyses of three-dimensional (3D) anatomical structures and quantified gene expression densities are critical because the structure is extremely complex and strongly related to its function, and activation or suppression of specific subsets of genes regulates cell-type or region-specific functions. As such, systematic approaches are needed to perform unbiased and comprehensive 3D mapping of gene expression densities in relation to anatomical structures.

Previous in situ hybridization (ISH)-based approaches to comprehensive endogenous gene expression (transcriptome) maps in the mouse include: the Edinburgh Mouse Atlas of Gene Expression (EMAGE) for the embryo equipped with section data, whole-mount data and optical projection tomography OPT data [4], [5], GenePaint [6], St. Jude’s Brain Gene Expression Map (BGEM) [7] and the Allen Brain Atlas of the Mouse (ABA) [3], which covers the adult whole mouse brain in 200 µm resolution of slice distances. In these databases, systematic investigation of fine expression data would be invaluable because gene expression is characterized at the cellular level. Histological analyses are rich in information in the x–y planes, but the information in the z axis is difficult to obtain at precisely the same level without extremely time-consuming and labor-intensive experimental efforts. Therefore, 3D reconstruction experiments have been limited to very selective conditions.

An alternative approach is a high-throughput analysis of gene expression data obtained by combination of microarrays or RNA-sequence methods and sample cubing. Voxelation Map [8], [9] and BrainStars [10] produce comprehensive expression datasets precise enough to recognize expression patterns in sub-structural regions of the macroscopic anatomy of the mouse brain at a resolution of 1 mm^3^ and in 500 µm-diameter regions, respectively. However, an increase in the resolution requires a cubic increase in sample numbers. Practically, about 50 data points were used for covering throughout the striatum level and for examining spotted areas mainly in the brainstem, respectively, in these studies; and the recently announced human whole brain atlas in ABA surveyed microarray with over 900 data points of dissected tissues. Therefore, investigations must be restricted to selected areas or low resolutions; otherwise, well-designed experimental systems are required to show a new paradigm for systemic approach to gene regulation for brain function.

Here we introduce a novel framework, Transcriptome Tomography, to create comprehensive expression maps in a broad and unbiased 3D-anatomical context using a relatively small number of original data points. The data were obtained from multiple samples sectioned in each of three orthogonal planes and were reconstructed into a 3D image space. This framework enabled us to rapidly produce an anatomical overview of comprehensive gene expression in the entire sample. We produced the first dataset of quantified expression densities in the mouse brain, including greater than 36,000 maps reconstructed from 61 microarray data of six mice placed in an anatomical context (ViBrism: http://vibrism.riken.jp/3dviewer/ex/index.html and examples to show how ViBrism-DB is to be used; Videos A–C in http://opensciences.brent-research.org/home/project-updates). After computational estimation of mapping accuracy, we evaluated the first dataset by comparative studies to existing datasets of ABA and BrainStars. The maps were isotropic and well suited in the recently proposed standard coordinate space for digital atlases: the Waxholm space [11]. We would demonstrate how this expression maps can be useful by assessing maps of Huntington’s disease related genes.

## Results

### Semi-automated Expression Mapping of Genes in a 3D Anatomical Context

**Figure 1:**
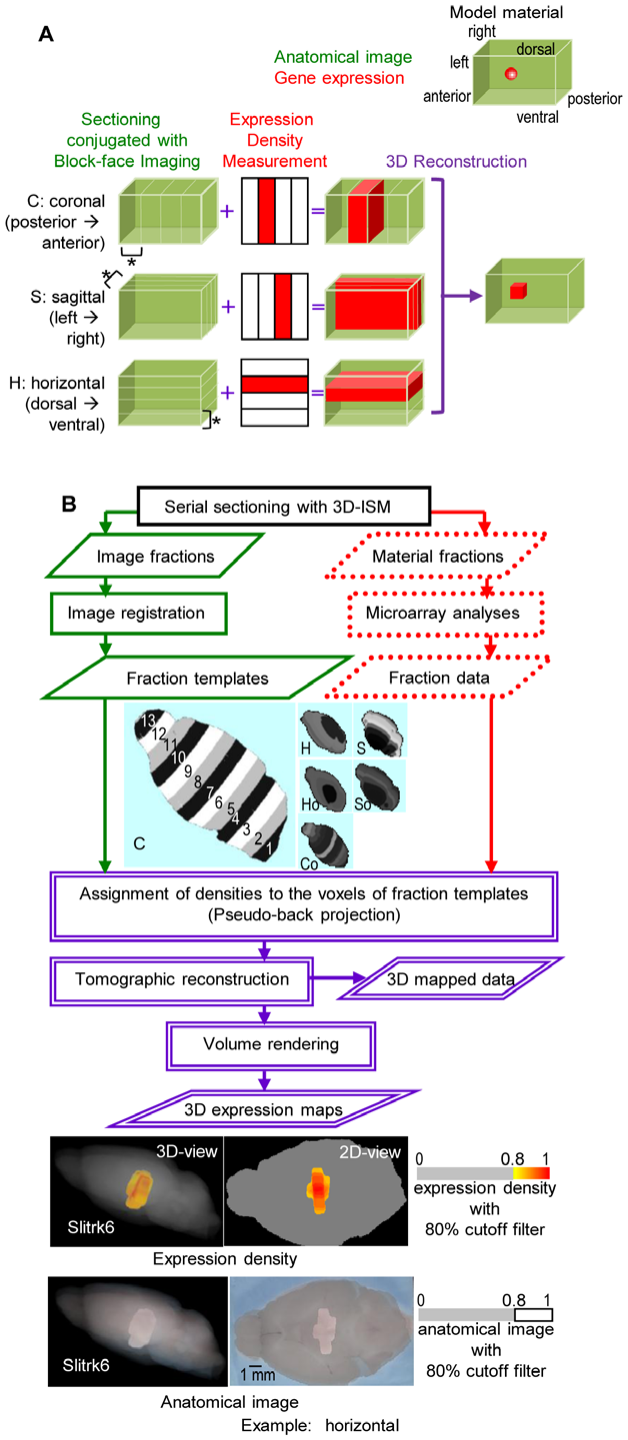
Transcriptome Tomography. (**A**) **A schematic illustrated using a model material.** Two types of data, material shape images (drawn with green lines) and gene expression densities (shown in red) of fractions (indicated with asterisks), are obtained with sectioning, conjugated with block-face imaging and expression density measurement, along three body axes (shown in parentheses). The three series of sectioning are named after orthogonal planes (C, S and H). The densities are assigned to the voxels (pixels on a regular grid in a 3D space) in the images (as shown with +) and subjected to tomographic reconstruction (indicated in purple). A series of the process from one direction needs one material; therefore, at least three genetically identical materials were required. (**B**) **An outline of the technique and the first dataset creation.** Two types of data, fraction templates, which are the material shape image (in green) and fraction data, which are gene expression densities measured with microarray (in dotted red), were acquired from the same fractions prepared with a sectioning machine 3D-ISM [12]. The fractions were named “image fractions” for the former data and “material fractions” for the latter (the preparation process seen in Video S1). Six fraction templates for the first dataset, two groups of three series sectioned in each of orthogonal and slightly oblique to the orthogonal planes: S/C/H and So/Co/Ho, composed of 9/13/6 and 10/16/7 fractions, respectively, (61 fractions in total as seen in Figure S1B), are shown with fraction numbers in Template C: 13 fractions of 1 mm (5 µm×200 sections)-thickness. The pseudo-tomography technique of mapping in a single coordinate space (named ViBrism) including image registration, pseudo-back projection and tomographic reconstruction is shown in the flowchart (see details in Figure S1A and Text S1). After volume rendering, 3D expression maps for genes (a sample: Slitrk6) are visualized as pseudo-colored expression densities and anatomical images with an 80% cutoff filter (also seen in Video S2). Slitrk6 is known to be expressed mostly in the thalamus as shown in the Allen Brain Atlas and BrainStars databases: 2Dand 3D views displayed here are compatible to those data shown below in Figure 4A and B and VideoS2.

Aiming at rapid and unbiased mapping of gene expression densities in a 3D image space, we introduce a tomographic framework, Transcriptome Tomography. Its technique is based on block-face imaging combined with tissue sectioning, which allows image reconstruction plus collection of tissues and expression analysis. To implement the tomographic approach to complex anatomical structures, we propose a novel framework for semi-automated sampling and mapping (Figure 1). Two types of data, non-stained tissue block-face images and gene expression densities measured with molecular methods, were acquired from a series of sliced tissues, which are termed a series of fractions. The data of multiple series obtained in multiple slicing directions, for instance three orthogonal sectioning planes (coronal, sagittal and horizontal), were then reconstructed into a single coordinate space as a 3D gene expression map using tomography techniques (Figure 1A).

In the first instance, we applied this framework to the adult mouse brain and created a dataset of 36,558 expression maps (Figure 1B). The data acquisition process using a sectioning machine, 3D-ISM [12], was as follows: a block-face image was obtained before each 5 µm section was cut, then a serial set of sections (200 sections) was collected in batches as a fraction (5 µm×200 = 1,000 µm in width) and a series of fractions was obtained using a whole brain (Video S1). One brain sample is needed for each of the series and therefore, multiple samples of genetically identical littermates were required for an isotropic 3D reconstruction. The first dataset was created using six brain samples (61 fractions of six series in total, seen in Figure S1B). Three series was the minimal requirement for 3D reconstruction. However, six brain samples were generated for the purpose of statistical analysis. Block-face image data of the six brains underwent an image registration process to produce fraction templates (Video S2) of the virtual brain, which was a single brain in an x-y-z coordinate space displaying anatomical information (ViBrism: virtual brain with 3D-ISM). Comprehensive gene expression density were measured as intensity values on the microarray experimental platform using 36,558 probes (total probes, definition seen in the Materials and Methods) in each of the 61 fractions (fraction data). Densities were assigned to voxels of the fraction templates. This process comparable to back-projection in tomography was followed by the 3D reconstruction of the expression maps by averaging the densities in the voxels (3D mapped data) and 3D expression map visualization (Video S2). It required about one month for the creation of 3D mapped data and 3D expression maps (see details in the Text S1 for Supporting Methods and Figure S1).

### Validation of the First Dataset

**Figure 2:**
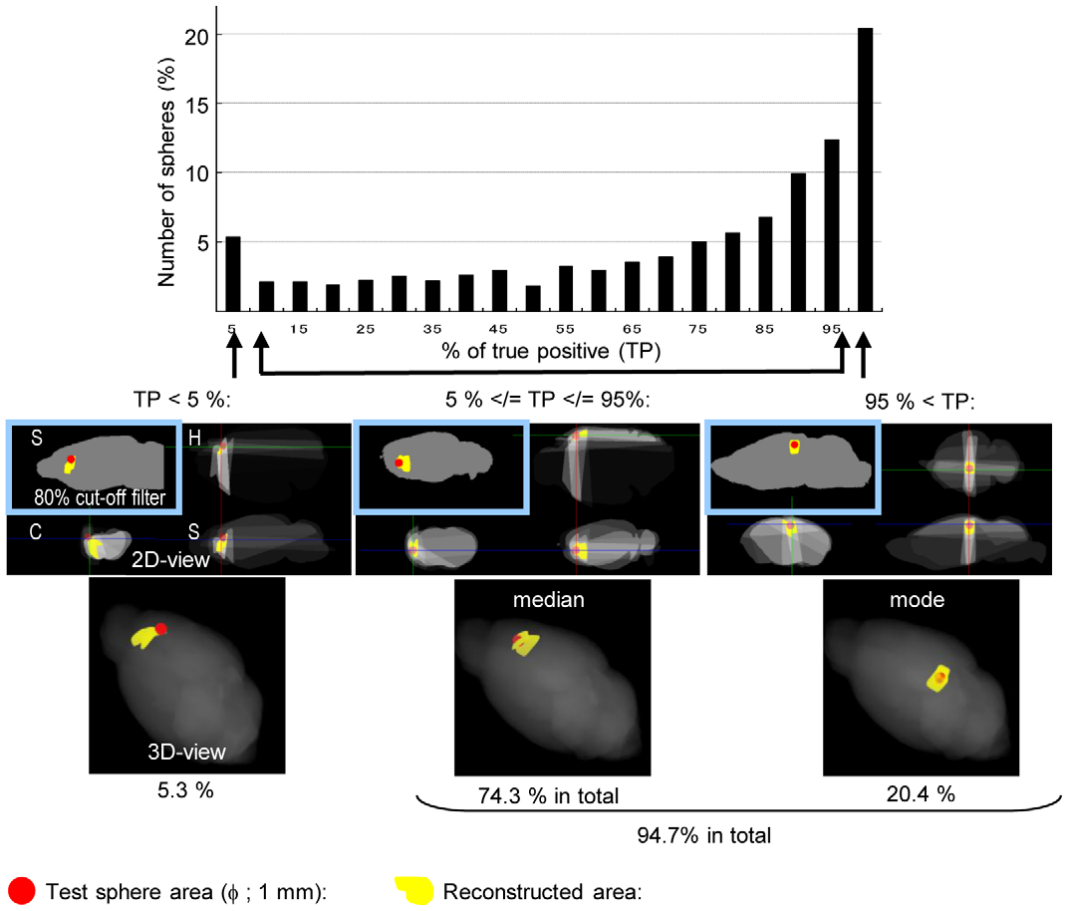
Results for the computational experiment of reconstruction using 1,366 test spheres. Gene expression that was evenly distributed in one of the test spheres located randomly in the virtual brain of ViBrism was computationally reconstructed (see Text S2 for Supporting Methods). A histogram for the number of test spheres with true positive rates (% of TP: percentages of test sphere volumes overlapped with the reconstructed area) is shown. Maps of the reconstructed results (shown in yellow) with the test spheres (in red) are attached. In 2D maps, the 80% cutoff filter was applied to the results of left-upper S panels; otherwise, the reconstructed densities are shown in gray scales. 3D maps are shown with the filter. Approximately one fifth (20.4%) of the test spheres had more than 95% of TP, which is the mode in the histogram, and 94.7% in total had at least 5% of TP as indicated. One of the mode results, the median result (TP = 80%) and one of the poorly reconstructed results (TP<5%) are shown. Only 0.8% of the test spheres resulted in no TP, which was mainly due to the peripheral location of the test spheres in the virtual brain (data not shown).

Prior to 3D mapping the biological experimental dataset, accuracy of the present tomography technique was computationally estimated using 1,366 test spheres, phantoms of gene expression randomly located in the ViBrism space, and the diameter was 1,000 µm (Figure 2). The expression areas were reconstructed using the same fraction templates as the first dataset (see Supporting Methods in Text S2). Approximately 95% of the reconstructed areas (1,293/1,366 areas) overlapped with at least 5% of the corresponding test sphere areas in volume, and the reconstructed area was 1.7-fold larger in diameter than the test sphere areas (1.73+/−0.01). These results demonstrate that our present techniques were sufficient to reconstruct a 1,000-µm-diameter object located in most areas of the brain despite the size-over estimation.

The fraction data, gene expression density data measured in each of the fractions as microarray intensity values using the total probes, were statistically validated. The unusual sectioning method to produce the fractions, which considered body axes but not anatomical regions, resulted in high correlation coefficients of the intensity values (0.976+/−1.90×10^−4^) between the fractions, and the coefficients ranged from 0.945 to 0.995. The lowest coefficient was observed between Co5 (the fifth fraction in Co) and So1, which shared little areas of the brain. Higher correlations were found between S1–4 and S9–6, respectively (0.988+/−0.019, p<1.5×10^−3^), in accordance with the adjusted axis of the S preparation toward anatomical symmetry. In addition, high correlations were found in paired fractions between the groups, S/C/H and So/Co/Ho (0.987+/−6.18×10^−4^, p<1.0×10^−12^, “slightly different” biological replicates: Table S1). These results demonstrate the reproducibility of our data; we found similar intensity value profiles for the total probes in the fractions derived from anatomically similar areas. Indeed, 3D-ISM could not produce brain fractions treated as exact biological replicates because of its mechanical sectioning nature. Therefore, the “slightly different” biological replicates would be used for further statistical analysis with variables (see the next section).

**Figure 3:**
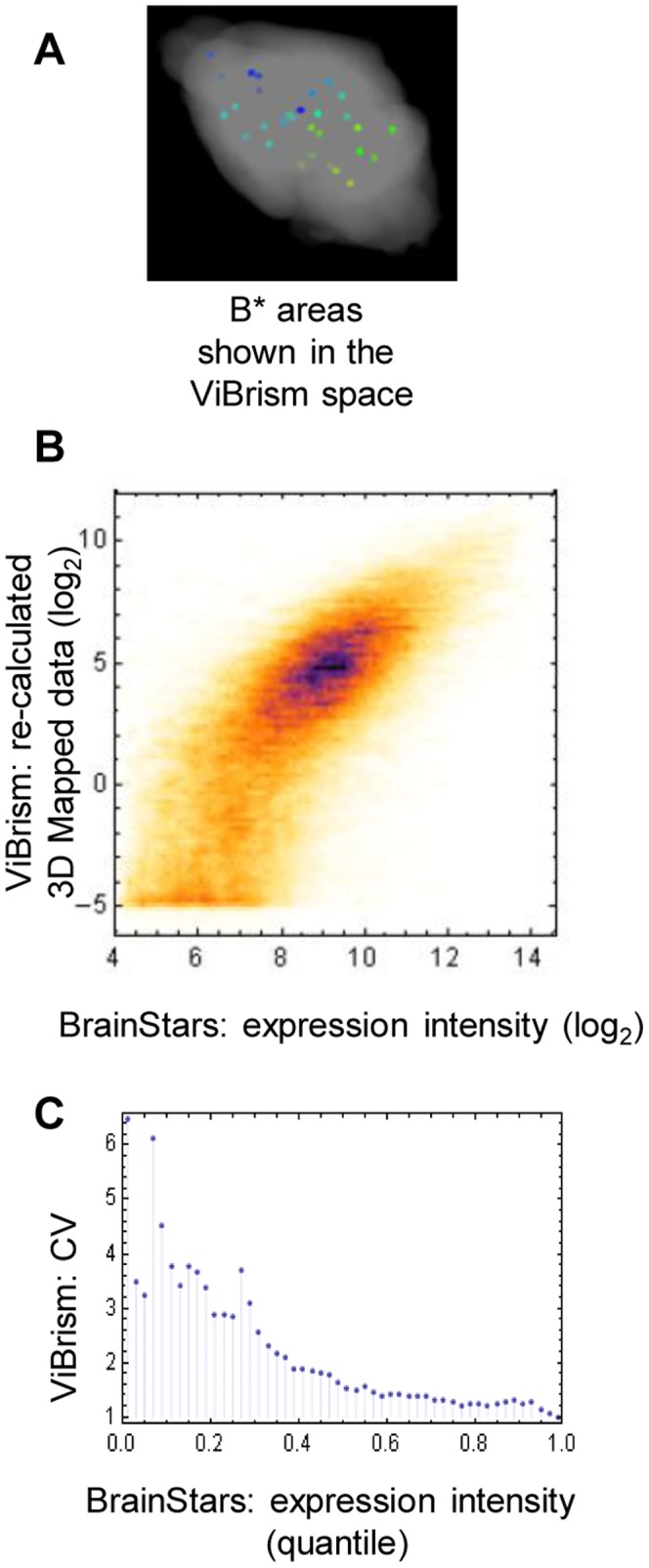
Validation of 3D mapped data. (**A**) B* areas mapped in the ViBrism space. To compare datasets obtained from different anatomical contexts, areas selected for BrainStars analysis (B* areas; 500-µm-diameter spheres) were mapped onto the ViBrism space as shown in the website Video C (http://opensciences.brent-research.org/home/project-updates). (**B**) Comparison of the expression datasets of ViBrism 3D mapped data and BrainStars data in B* areas in a scatter plot. 3D mapped data re-calculated in the 46 areas for 17,155 gene probes were log-2 transformed and plotted against BrainStars expression intensity data. Despite two datasets were obtained from different anatomical contexts using different material preparation procedures and microarray platforms, good agreement was observed in a scatter plot (r = 0.73, p<1.0×10^−12^). (**C**) **Coefficient of variance (CV) of the 3D-mapped data for each quantile window of the corresponding expression intensity values in the BrainStars dataset.** For the each 0.02-quantile window of the BrainStars dataset, the CVs (standard deviation/mean of non-log transformed values) of the re-calculated 3D-mapped data were calculated and dotted.

To validate the 3D mapped data, we compared them with expression data of BrainStars, in which the microarray dataset is created at 500-µm resolution [10]. Common expression density data of 17,155 probes were available in 46 areas of spheres (B* areas, Table S2). We first identified the centroids of the areas in the block-face images of the anatomical image atlas in the ViBrism space (the atlas shown in Video S2), and labeled the spheres on the space (Figure 3A; see the procedure in Materials and Methods, and the website Video C: http://opensciences.brent-research.org/home/project-updates). Mean values of 3D mapped data in the area were calculated for each of the probes (re-calculated 3D mapped data) and compared to the BrainStars dataset. Considering the previous inter-platform comparison study of microarray datasets [13], good agreement was observed between the two datasets in a scatter plot (Figure 3B, r = 0.73, p<1.0×10^−12^). Coefficient of variation (CV) of the re-calculated 3D mapped data for each 0.02-quantile window of the expression intensity values of the corresponding BrainStars dataset showed a large extent of variation normalized to the mean particularly in low expressed genes (<0.4-quantile, Figure 3C). These results indicate the overall relevance of our computationally mapped data to the manually-prepared data with respect to the location and density of gene expression; and the accuracy of the mapped expression density depending on the sensitivity of the measurement of expression density.

**Figure 4:**
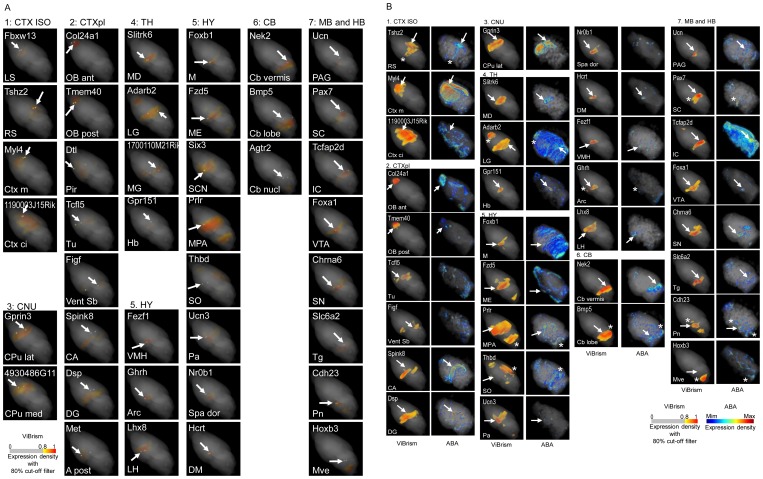
Validation of 3D expression maps. (**A**) Comparison of 3D expression maps to B* area maps. 3D expression maps of 40 regionally expressed genes were chosen (Table S2). Corresponding B* area maps were overlaid on the expression maps and highlighted as 500 µm in diameters with VCAT. The names of the genes and the B* areas are shown at the top and the bottom of panels respectively. Spheres of the B* areas are indicated with arrows in the panels. If the gene expression areas indicated with yellow/orange/red colors are located in the B* areas, the colors are highlighted to be seen in the spheres (23 genes): otherwise, gray colors appear. In most latter cases, the gene expression areas were located close to the highlighted spheres, suggesting that the distances between the areas and the spheres were mainly caused by the resolution differences of two systems. All maps were viewed in the same angle to show the expression pattern differences. Color codes for are shown. (**B**) **Comparison of 3D Expression maps to ABA maps.** Among the 40 regionally expressed genes, 3D map data of 33 genes were available in ABA. Expression maps in ABA and our maps (ViBrism) were visualized with VCAT. The names of the gene and the B* area, in which the gene was selectively expressed, are shown at the top and the bottom of panels for ViBrism, respectively. B* areas are indicated with arrows in the maps of ViBrism in the same way as Figure 4A and are also indicated in the maps of ABA if the areas were visualized with high expression. Expression areas common in the two maps but outside the named B* areas are indicated with asterisks. All maps are shown in the same angle to show the expression pattern differences. Color codes for ABA and ViBrism are shown.

To validate the 3D expression maps, data for 40 regionally expressed genes nominated through the BrainStars experiment (Table S2) were compared among the three datasets of ABA, BrainStars and ViBrism. We examined whether areas with gene expression in the 3D expression maps included corresponding B* areas and whether our maps were consistent with the ABA 3D maps (Figure 4A and B). Most of the genes were mapped in comparable or closely located anatomical regions in the three datasets. Although the similarities between our dataset and the ABA’s dataset were unquantifiable because of anatomical context differences, the genes in a variety of areas could be mapped and showed at least 40 different expression patterns that were distinguishable in our maps.

### Fraction Data Analysis with Variables

To characterize the fraction data independently from mapping, we calculated sets of variables, *I* and *V*, for each probe in the fractions. *I* was a set of median expression intensity values. *V* was defined as a set of false discovery rates (FDRs) of expression variance calculated with an ANOVA (see the Materials and Methods). Variables *I* and *V* were independent (r = −0.02), and the total probes were categorized into the groups, IV, iV, Iv and iv (12,061, 9,927, 6,218 and 8,352 probes, respectively). Group I had a higher median intensity values than the median of the total probes, and group V had a statistically significant expression variance (non-uniform expression, FDR<0.05). Among the 784 regionally expressed genes selected with ISH methods in the ABA and the 240 genes with cell type-specific expression selected with molecular methods [3], [14], 83.2% and 84.9% of the genes belonged to group V, respectively, though our results with present resolution showed less significant levels of variance in anatomical expression location. The genes with strictly even expression [14] belonged to group Iv, as expected. These results showed sufficient consistency between the fraction data and previous results with respect to expression variances, despite the difference in analytical methods.

### Analysis of a Neurodegenerative Disease-causing Gene, Huntingtin (*Htt)*

We assessed whether our analysis framework could reveal new insight in a disease-related gene expression. As an example, Huntington’s disease gene (*Htt*) [15] of the mouse was examined (also called *Hdh* in *Mus musculus*). This genetic disease is characterized by pathological findings of increased vulnerability of medium spiny neurons in the brain region, caudate putamen (CPu). These neurons are specified by receptors and their downstream signaling molecules for brain derived neurotrophic factor (Bdnf) or Dopamine as well as by the production of neurotransmitter GABA. As the disease progresses, the pathologies progressively extend and a significant loss of neurons is observed in other brain regions such as the cerebral cortex. Whereas, *Htt* gene products are present in most cells with no evidence of increased normal or abnormal gene products in the affected brain regions of the human or mice [16], [17].

**Figure 5:**
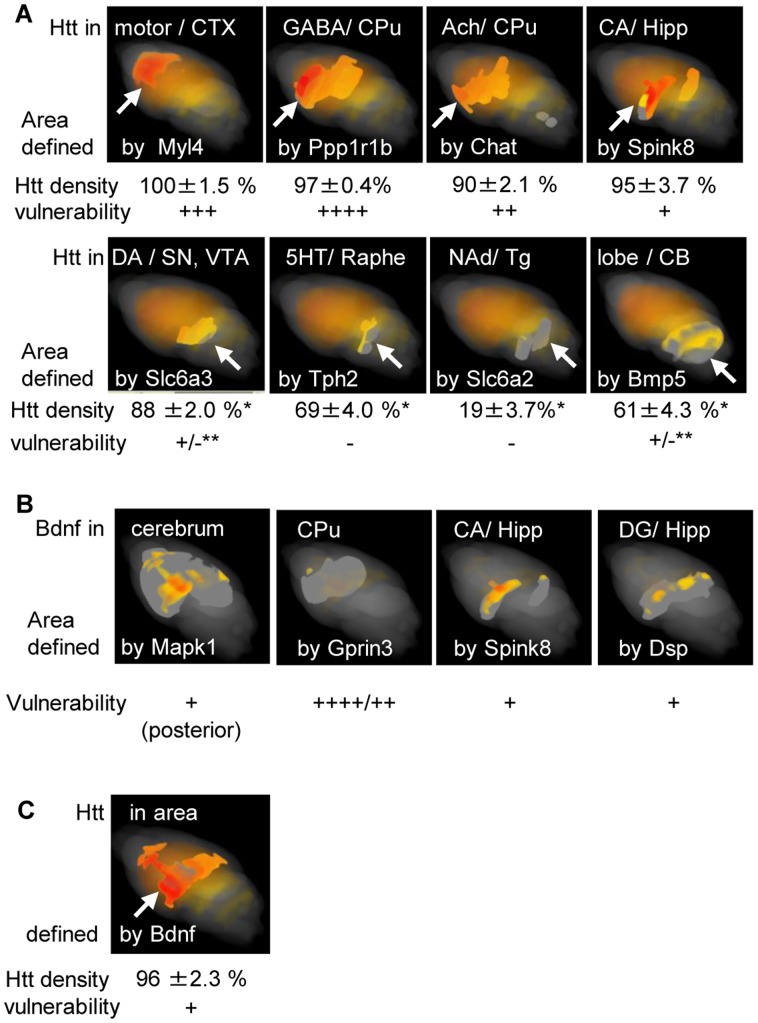
Expression densities of Huntington’s disease related genes in the brain areas defined by area-marker gene expression. **(A) Quantified expression variances of** ***Htt*** **, related to differential vulnerability in the disease.** 3D expression maps of *Htt* are highlighted in areas (with arrows) defined by the expression of area-marker genes (named at the bottom of the panels), which are known to be expressed in the anatomical regions or the cell types (named at the top). Percentages of *Htt* expression densities (100% in the motor CTX) and the vulnerability of the areas in Huntington’s disease are indicated below the panels. *: significantly lower compared to motor CTX (p = 10^−5^–10^−41^), **: selective cells are partly affected [21] [23]. (**B**) **Expression variances of Bdnf related to the vulnerability of the disease.** 3D expression maps of *Bdnf* in the areas defined by the expression of area maker genes as indicated are shown along with the vulnerability of the areas. (**C**) **Expression maps of** ***Htt*** **in brain areas defined by** ***Bdnf*** **expression.** The maps, a percentage of *Htt* expression densities and the vulnerability of the areas are visualized in the same way as in A. An Arrow indicates high expression of *Htt*, and *Bdnf* in the posterior cerebrum. Abbreviations for neural cell types: GABA: GABAergic, Ach: Acetyl-cholinergic, DA: dopaminergic, 5HT: serotonergic, NAd: noradrenalinergic. Abbreviations for brain regions: CA: Ammon’s Horn, CB: cerebellum, CPu: caudate putamen, CTX: cerebral cortex, Hipp: hippocampus, DG: dentate gyrus, SN: substantia nigra, VTA: ventral tegmental area, Tg: dorsal tegmental nucleus.

In this study, *Htt* exhibited a statistically significant variance of expression (FDR = 0.023). To characterize the variance, we first defined the brain areas of interest, in which differential vulnerabilities have been reported, using 3D expression maps of area-marker genes, the regionally expressed genes nominated in Table S2 or previously known [17]–[20]: and then we measured the expression density of *Htt* in these areas (Figure 5A). The densities in the different areas varied (p<10^−15^). Particularly densities in the areas with decreased vulnerability, such as the SN/VTA, raphe nuclei, Tg and CB lobe[21]–[23] were significantly lower than in the motor cortex (p = 10^−5^–10^−41^); non-uniform expression of *Htt* was also observed in the predetermined ABA regions http://mouse.brain-map.org/brain/gene/69734971/ExpressionGraph.html: our analysis, which defined brain areas by marker gene expression, showed a statistically significant expression variances of *Htt* compatible to the differential vulnerability.

Moreover, we analyzed the disease related *Bdnf* gene, a decrease in which is strongly related to Huntington’s disease pathology [24]. Here, we demonstrate that *Bdnf* was non-uniformly expressed (FDR = 8.93×10^−8^) with relative expression densities comparable to vulnerability of the brain areas: low expression in the CPu, the most vulnerable region in this disease, and high in the posterior cerebrum including the hippocampus, the less vulnerable region (Figure 5B). The high expression of neurotrophic *Bdnf* may account for the less disease pathology of the posterior cerebrum in Huntington’s disease [25], despite the high expression of *Htt* (Figure 5C).Our analysis revealed a statistically significant expression variances of the disease related genes comparable to the differential vulnerability, presuming biological significances of their combinatorial expression in the characteristic pathology.

### Spatial Integration of the Maps into the 3D Standard Coordinate Space for Digital Brain Atlases, Waxholm Space (WHS)

The transcriptome 3D map dataset was rapidly produced and also unbiased because the measurement and reconstruction of expression densities were based directly on volume and not on many pre-set planes. Consequently, anatomical accuracy was spatially isotropic, in contrast to section-based ISH data with high resolution only in the sectioning planes.

**Figure 6:**
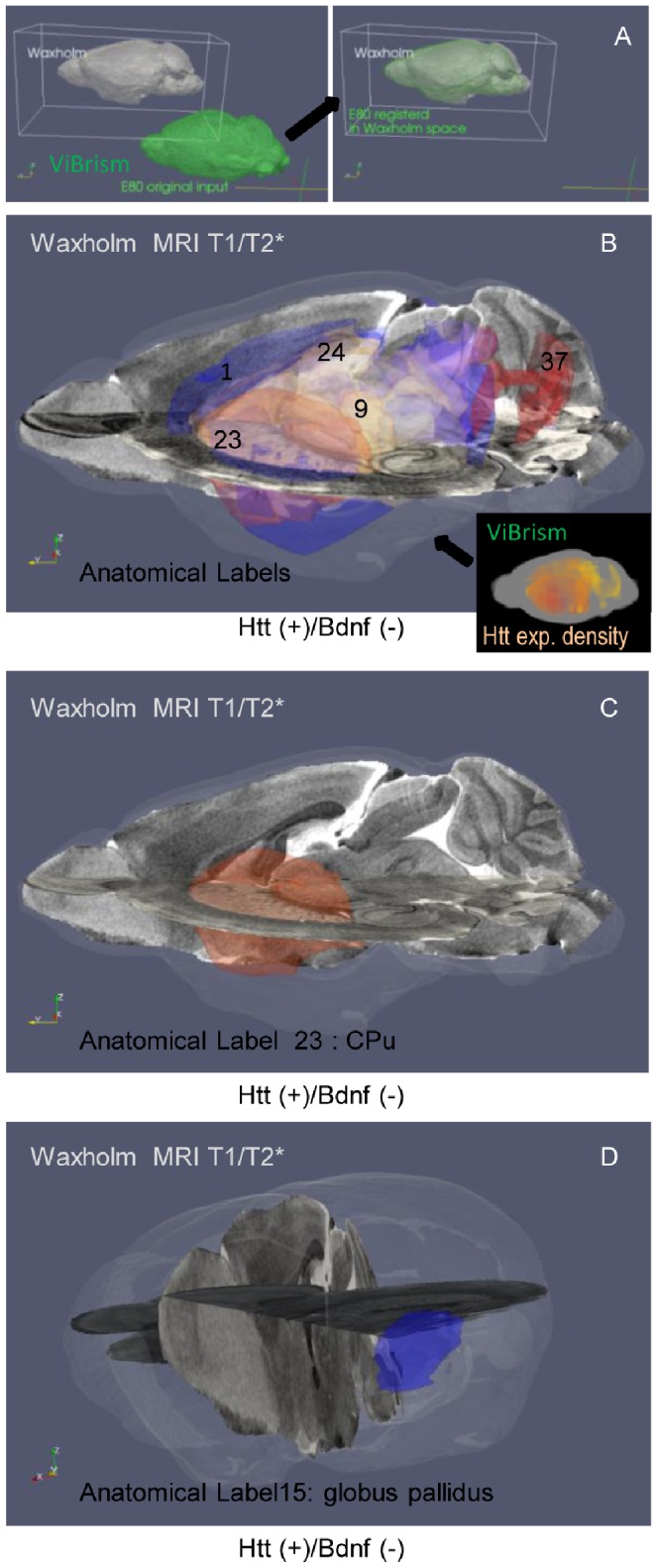
Spatial integration of 3D expression maps into the WHS MRI digital atlas. (**A**) **A schematic for the integration.** E80, the brain volume common to all expression maps in the ViBrism space, (colored in green) was transformed (shown with an arrow) into the brain volume in the Waxholm Space (WHS in gray). (**B–D**) **Integration of Huntington’s disease-related maps.** Areas defined by the gene expression of a pathogenetic combination deduced from the previous knowledge, Htt(+)/Bdnf(−), are colored with the Htt expression density in the ViBrism space (a right small panel in B). After the transformation into WHS, the areas are highlighted and colored with anatomical labels based on MRI data [26] (a large panel in B). The areas contain vulnerable regions in this disease as follows: Label 1; Cerebral cortex (anterior rather than posterior, 22% of the volume with label 1), 9; Ventral thalamic nuclei (100%) [28], 15; Globus pallidus (100%) [17] shown in the panel D, 23; CPu (99%) also seen in the panel C, 24; hippocampus (77%), 37; cerebellum (21%). Table S3 shows % of volumes overlapped with the Htt(+)Bdnf(−) areas in each of 37 anatomical regions labeled in WHS. The MRI T1 and T2* atlases are shown in the rectangular vertical and horizontal planes, respectively.

Therefore, we can simply integrate the 3D expression maps into standard coordinate space for digital brain atlases, Waxholm Space (WHS) [11], [26], using a single automated pipeline composed of web-accessible computational programs [27]. To show an advantage of the spatial integration of multiple maps into the space, the brain areas defined by the expression of *Htt*(+)/*Bdnf*(−), which we expected to be highly vulnerable in Huntington’s disease as mentioned in the previous section, were overlaid onto the MRI atlases in WHS and highlighted with colors representing the anatomical regions based on MRI data [26]. These regions, 39% of the whole brain volume, were highlighted (Figure 6, Table S3) and indeed, contained highly vulnerable regions in Huntington’s disease, 99% volumes of the CPu and 100% volumes of the ventral thalamic nuclei and the globus pallidus [28], [29], but did not contain regions with minimal or no pathological findings such as the pontine gray and the pineal grand. Volume percent of the brain regions involved in the *Htt*(+)/*Bdnf*(−) area in each of the 37 anatomical regions in WHS seemed roughly correlated to the disease vulnerability (Table S3). Here we demonstrate a potential use of spatial integration in identifying brain regions defined by gene expression in ViBrism space as anatomical regions in the whole brain context of WHS, on which broad information is available. This allows for correlation between the ViBrism dataset and other WHS datasets that use this spatial framework.

## Discussion

We report on a method for tomographic acquisition of comprehensive gene expression data. Transcriptome Tomography is a form of sample pooling, producing materials of a variety of mixed cell types, which is statistically valid with equivalent power to a modest increase in sample number for analysis, and the power depends on the sensitivity of measurement system [30]. We consider this a time-and-cost-effective strategy for acquiring comprehensive gene expression data in a spatial context. The mapping accuracy of the tomography technique depends on the number of data points [31]. These are dependent on the fraction width and the number of sectioning directions, such that by increasing the points over the brain regions one increases the regional accuracy. In the present dataset, there are sufficient data points to show spatial distribution of gene expression in anatomical sub-regions. However, very selective expression, for instance, in a layer of a part of the cortex, must be un-mapped or over-estimated because of the measurement sensitivity or the present fraction width, respectively. Biologically, one twentieth of the present width, a 50-µm-thick fraction, is sufficient to obtain RNA materials for an analysis in the present molecular measurement technique. Therefore, isotropic maps of higher resolution than existing ABA (200 micron at a distance in the sagittal plane) can be produced in a cost-effective manner within remarkably less time using the Transcriptome Tomography approach. We envisage this framework being particularly useful in studies which require multiple comprehensive datasets to be compared, for instance, genetic mutant analyses, pharmacological studies and cross-age comparisons.

In this study, a small number of brains were examined with simple semi-automated molecular procedures, and expression densities were measured with about 37,000 probes on one microarray platform that was designed to detect most of coding genes. Therefore, the resulting data can be compared with one another without considering experimental variations for each gene and 3D expression maps were used to quantitatively assess gene expression patterns. By comparison, ISH shows cellular expression profile for each gene on a slice plane. However, it is very difficult to normalize and compare expression densities of thousands of different genes on a similar slice plane. Transcriptome Tomography enables us to display comparative expression densities of multiple genes on sagittal, coronal, or any directional section planes (seen in the website Video B: http://opensciences.brent-research.org/home/project-updates ) and in any areas. We used this strategy to quantify expression densities of Huntington’s disease related genes. An average expression density of a gene in an area is related to the expressing cell density and the expression amount per cell. Htt is expressed in many types of cells and expression variance we observed may reflect the diverse pattern of expressing cells, which may ultimately relate to disease vulnerability in specific brain regions. We envisage that an approach that seeks to integrate data derived from cell-based analyses such as ISH and from the volume-based tomographic framework into the common 3D space would be extremely useful for interpretation of expression diversities.

The standard space for multiple expression data is particularly important for systematic approaches to the brain biology: its significance is analogous to encoded standard sequences for genome biology. Our framework for time-and-cost-effective mapping will facilitate researchers to create their own datasets in many experimental conditions and to analyze them in the standard space. In addition, the tomographic approach introduced here is applicable to analysis of any bio-molecules, proteins, lipids and sugars, in addition to a variety of RNA forms such as microRNAs or long non-coding RNAs measurable on new microarray platforms or RNA sequencing, extracted from any frozen tissues, organs and whole embryos. We hope that our framework will contribute to a progress of molecular expression based systemic approaches to complex structures and function and the abnormalities.

## Materials and Methods

### Ethics Statement

All procedures involving animals and their care were performed according to the RIKEN Regulations for Animal Experiments (approval ID: H19-1W009).

### Brain Sample Preparation

Preparation methods are described in the Text S1 for Supporting Methods. Briefly, frozen brain samples were obtained from 8-week-old male C57BL/6J mice (Japan SLC), the cross-sectioning planes (5 µm) were sequentially produced and their block-face images were obtained using a sectioning machine 3D-ISM [12] to visualize the 3D anatomical context of the brain. 1-mm (5 µm×200 sections in a batch)-thick fractions (material fractions) were collected and used for microarray analyses. This process was performed in six mice and resulted in six series of sectioning for a total of 61 material fractions (as shown in Figure S1B).

### Microarray Procedures

The 61 material fractions were kept frozen in 200 µl of TRI reagent (Ambion). A portion (500 ng) of total RNA extracted from the fraction, treated with Mag-max-96 for Microarrays Total RNA Isolation Kit (Ambion), was used in the microarray experiment with the standard single color protocol (Whole Mouse Genome 012694, Agilent) [32]. Measured intensity values on the microarray platform were per-chip normalized with GeneSpring GX software. Gene probes with at least one ‘present’ flag calling were denoted as total probes (36,558 probes), and their data in the 61 fractions were subjected to the following studies (fraction data).Those probes are enough to detect most coding genes (ca. 25,000 genes) and splice variants. The microarray data discussed in this publication have been deposited in NCBI’s Gene Expression Omnibus [33] and are accessible through GEO Series accession number GSE36408 (http://www.ncbi.nlm.nih.gov/geo/query/acc.cgi?acc=GSE36408).

### Validation of 3D Mapped Data and 3D Expression Maps

A dataset of BrainStars was retrieved from the database (http://brainstars.org/). To compare the gene expression data produced in the two microarray platforms, the probe with the highest mean expression intensity values of a gene in each platform (17,155 probes) was selected. To produce anatomically comparable data, we identified the centroids of the B* areas bilaterally in the Anatomical Image Atlas with eyes of anatomists, which was done exactly in the same way, but virtually (see the website Video C: http://opensciences.brent-research.org/home/project-updates), as done for the real mouse brain using the previously described criteria [10], and then labeled the area as 500-µm-diameter spheres using a visualization software, VCAT. Mean values of 3D mapped data without per-gene normalization and conversion to 8-bit intensity grades were calculated in the labeled areas for each of the 17,155 probes, and bilaterally averaged (re-calculated 3D mapped data). The coefficient of variation of the re-calculated 3D mapped data for each 0.02-quantile window of the expression intensity values of the corresponding genes and brain areas in the BrainStars dataset were obtained by trimming 0.1% from the both ends of the ViBrism expression values in the window followed by calculating standard deviation/mean of the trimmed ones.

A digital map dataset of ABA were retrieved from the database of Allen Institute for Brain Science (http://www.brain-map.org/), converted to VCAT files and visualized with 256 color codes. The regionally expressed genes were selected from “marker gene candidates” in the BrainStars database (http://brainstars.org/marker/).

### Fraction Data Analyses Using Variables

Two sets of variables, *I* and *V* were calculated in log-transformed microarray intensity values of fraction data. The variable *I* represented the intensity medians for the probes in the 61 fractions. The variable *V*, the variance of intensity for the probe in the fractions, was defined as FDR: rate of false discovery of uniformly expressed genes as non-uniformly expressed genes, calculated using a one-way ANOVA with multiple-testing correction of Benjamini and Hochberg. 3D-ISM could not produce brain fractions treated as exact biological replicates. Therefore, “slightly different” biological replicates were defined as follows and used for ANOVA (Table S1). Pearson correlation coefficients (r) of the microarray intensity values of the total probes between the fractions were calculated and used for correlation measures of a pairwise comparison between fractions in the two groups S/C/H and So/Co/Ho, and the pairs with the highest r were selected as the replicates. When multiple fractions in one group showed the highest r to a fraction in the other, the anatomical fraction order took precedence.

### Defining Brain Areas by Marker Gene Expression and Measuring Expression Density

The instruction for visualization of defined areas in 3D expression maps is shown in the Quick Manual for VCAT (downloadable from http://vibrism.riken.jp/quick_manual.pdf). The gene expression densities in the defined area were calculated as the averaged intensity values in the voxels of the area: a total of the 3D mapped data above the threshold value of the 80% cutoff filter in the voxels of the area was divided by the volume of the area.

### Integration of Maps to the Waxholm Space, WHS

The 3D expression maps, obtained by stacking the already co-aligned 2D sections, were deformably registered into alignment with the canonical T1 MRI volume of WHS. The open-source ANTS methods [27] used for the registration were incorporated within a processing pipeline specialized for the WHS normalization task. The resultant normalization transformations allow bi-directional transfer of information between the ViBrism space and WHS.

### Statistics

The methods for fraction data analyses were described above. Other methods used were as follows. The results are represented as the mean +/− s.e.m., r was calculated and used for correlation measures between two datasets and two-sided p-values calculated with Student’s *t*-test are shown. Alpha levels are 0.01 in the all analyses.

## Supporting Information

Figure S1 **The framework for 3D mapping of transcriptome and analyses.** **(A) A flowchart of the processes for the framework.** The steps of the processes are illustrated with shapes and colors. Legends for the shapes are in the right white panel and the colors are same as in Figure 1, with the light-blue color representing common processes. The processes are numbered and described in the Text S1. 3D-ISM was originally a device for sequential photography and was composed of three units: the image data acquisition device (indicated in green), the sectioning machine (in red) and the unit for synchronized sample feeding and blade rotation (in light-blue). A sample collection hole indicated by a yellow arrow is added to collect frozen sliced sections in batches (material fractions). **(B) Body axes-based sections and the number of fractions.** Arrows indicate the directions of the body axes-based sections of the model brain. The sectioning was performed in two groups of three series sectioned in each of orthogonal and slightly oblique to the orthogonal planes: S/C/H and So/Co/Ho, composed of 9/13/6 and 10/16/7 fractions, respectively, (61 fractions in total).
(TIF)

Table S1 **Biological replicates.**
(XLS)

Table S2 B* areas and regionally expressed genes.
(XLS)

Table S3 **Labels for anatomical regions in WHS and their volumes overlapped with Htt(+)Bdnf(−) areas.**
(XLS)

Text S1 **Supporting methods for Transcriptome Tomography.**
(DOC)

Text S2 **Supporting methods for accuracy estimation of the present tomography technique with a computational experiment using test spheres.**
(DOC)

Video S1 **Semi-automated sample sectioning with 3D-ISM.**
(WMV)

Video S2 **Image processing for 3D expression mapping.**
(WMV)

## References

[1] Stuart JM, Segal E, Koller D, Kim SK (2003) A gene-coexpression network for global discovery of conserved genetic modules. Science 302: 249–255 doi:10.1126/science.1087447. 12934013 10.1126/science.1087447

[2] Hobert O (2008) Regulatory logic of neuronal diversity: terminal selector genes and selector motifs. Proc Natl Acad Sci USA 105: 20067–20071 doi:10.1073/pnas.0806070105. 19104055 10.1073/pnas.0806070105 PMC2629285

[3] Lein ES, Hawrylycz MJ, Ao N, Ayres M, Bensinger A, et al (2007) Genome-wide atlas of gene expression in the adult mouse brain. Nature 445: 168–176 doi:10.1038/nature05453. 17151600 10.1038/nature05453

[4] Baldock RA, Bard JBL, Burger A, Burton N, Christiansen J, et al (2003) EMAP and EMAGE: a framework for understanding spatially organized data. Neuroinformatics 1: 309–325 doi:10.1385/NI:1:4: 309. 15043218 10.1385/NI:1:4:309

[5] Sharpe J, Ahlgren U, Perry P, Hill B, Ross A, et al (2002) Optical projection tomography as a tool for 3D microscopy and gene expression studies. Science 296: 541–545 doi:10.1126/science.1068206. 11964482 10.1126/science.1068206

[6] Visel A, Thaller C, Eichele G (2004) GenePaint.org: an atlas of gene expression patterns in the mouse embryo. Nucleic Acids Res 32: D552–6 doi:10.1093/nar/gkh029. 14681479 10.1093/nar/gkh029 PMC308763

[7] Magdaleno S, Jensen P, Brumwell CL, Seal A, Lehman K, et al (2006) BGEM: an in situ hybridization database of gene expression in the embryonic and adult mouse nervous system. PLoS Biol 4: e86 doi:10.1371/journal.pbio.0040086. 16602821 10.1371/journal.pbio.0040086 PMC1413568

[8] Chin MH, Geng AB, Khan AH, Qian W-J, Petyuk VA, et al (2007) A genome-scale map of expression for a mouse brain section obtained using voxelation. Physiol Genomics 30: 313–321 doi:10.1152/physiolgenomics.00287.2006. 17504947 10.1152/physiolgenomics.00287.2006 PMC3299369

[9] Singh RP, Brown VM, Chaudhari A, Khan AH, Ossadtchi A, et al.. (2003) High-resolution voxelation mapping of human and rodent brain gene expression. J Neurosci Methods 125: 93–101. PMID:12763235. 10.1016/s0165-0270(03)00045-1 12763235

[10] Kasukawa T, Masumoto K-H, Nikaido I, Nagano M, Uno KD, et al (2011) Quantitative expression profile of distinct functional regions in the adult mouse brain. PloS one 6: e23228 doi:10.1371/journal.pone.0023228. 21858037 10.1371/journal.pone.0023228 PMC3155528

[11] Hawrylycz M, Baldock RA, Burger A, Hashikawa T, Johnson GA, et al.. (2011) Digital atlasing and standardization in the mouse brain. PLoS ComputBiol 7: e1001065–. doi:10.1371/journal.pcbi.1001065.g006. 10.1371/journal.pcbi.1001065 PMC3033370 21304938

[12] Yokota H, Nakamura S, Kawaguchi R, Makunouchi A, Yabe H, et al.. (2002) Destructed Imaging of Biological Sample Using a 3 Dimensional Internal Structure Microscope. Medical Imaging Technology 20: 660–665. NAID: 10010350948.

[13] Shi L, Reid LH, Jones WD, Shippy R, Warrington JA, et al (2006) The MicroArray Quality Control (MAQC) project shows inter- and intraplatform reproducibility of gene expression measurements. Nature biotechnology 24: 1151–1161 doi:10.1038/nbt1239. 10.1038/nbt1239 PMC3272078 16964229

[14] Cahoy JD, Emery B, Kaushal A, Foo LC, Zamanian JL, et al (2008) A transcriptome database for astrocytes, neurons, and oligodendrocytes: a new resource for understanding brain development and function. J Neurosci 28: 264–278 doi:10.1523/JNEUROSCI.4178-07.2008. 18171944 10.1523/JNEUROSCI.4178-07.2008 PMC6671143

[15] MacDonald ME, Ambrose CM, Duyao MP, Myers RH, Lin C, et al (1993) A novel gene containing a trinucleotide repeat that is expanded and unstable on Huntington’s disease chromosomes. The Huntington’s Disease Collaborative Research Group. Cell 72: 971–983 doi:10.1016/0092-8674(93)90585-E. 8458085 10.1016/0092-8674(93)90585-e

[16] Walker FO (2007) Huntington’s disease. Lancet 369: 218–228 doi:10.1016/S0140-6736(07)60111-1. 17240289 10.1016/S0140-6736(07)60111-1

[17] Han I, You Y, Kordower JH, Brady ST, Morfini GA (2010) Differential vulnerability of neurons in Huntington’s disease: the role of cell type-specific features. J Neurochem 113: 1073–1091 doi:10.1111/j.1471-4159.2010.06672.x. 20236390 10.1111/j.1471-4159.2010.06672.x PMC2890032

[18] Li Y, Holtzman DM, Kromer LF, Kaplan DR, Chua-Couzens J, et al.. (1995) Regulation of TrkA and ChAT expression in developing rat basal forebrain: evidence that both exogenous and endogenous NGF regulate differentiation of cholinergic neurons. J Neurosci 15: 2888–2905. PMID: 7536822. 10.1523/JNEUROSCI.15-04-02888.1995 PMC6577746 7536822

[19] Liu Z, Yan SF, Walker JR, Zwingman TA, Jiang T, et al (2007) Study of gene function based on spatial co-expression in a high-resolution mouse brain atlas. BMC Syst Biol 1: 19 doi:10.1186/1752-0509-1-19. 17437647 10.1186/1752-0509-1-19 PMC1863433

[20] Walther DJ, Peter J-U, Bashammakh S, Hörtnagl H, Voits M, et al (2003) Synthesis of serotonin by a second tryptophan hydroxylase isoform. Science 299: 76 doi:10.1126/science.1078197. 12511643 10.1126/science.1078197

[21] Oyanagi K, Takeda S, Takahashi H, Ohama E, Ikuta F (1989) A quantitative investigation of the substantia nigra in Huntington’s disease. Ann Neurol 26: 13–19 doi:10.1002/ana.410260103. 2528318 10.1002/ana.410260103

[22] Heinsen H, Strik M, Bauer M, Luther K, Ulmar G, et al (1994) Cortical and striatal neurone number in Huntington’s disease. Acta Neuropathologica 88: 320–333 doi:10.1007/BF00310376. 7839825 10.1007/BF00310376

[23] Jeste DV, Barban L, Parisi J (1984) Reduced Purkinje cell density in Huntington’s disease. ExpNeurol 85: 78–86. PMID: 6203775. 10.1016/0014-4886(84)90162-6 6203775

[24] Zuccato C, Cattaneo E (2007) Role of brain-derived neurotrophic factor in Huntington’s disease. Progress in Neurobiology 81: 294–330 doi:10.1016/j.pneurobio.2007.01.003. 17379385 10.1016/j.pneurobio.2007.01.003

[25] Mann DMA, Oliver R, Snowden JS (1993) The topographic distribution of brain atrophy in Huntington’s disease and progressive supranuclear palsy. Acta Neuropathologica 85: 553–559 doi:10.1007/BF00230496. 8493863 10.1007/BF00230496

[26] Johnson GA, Badea A, Brandenburg J, Cofer G, Fubara B, et al (2010) Waxholm space: an image-based reference for coordinating mouse brain research. NeuroImage 53: 365–372 doi:10.1016/j.neuroimage.2010.06.067. 20600960 10.1016/j.neuroimage.2010.06.067 PMC2930145

[27] Avants BB, Tustison NJ, Song G, Cook PA, Klein A, et al (2011) A reproducible evaluation of ANTs similarity metric performance in brain image registration. NeuroImage 54: 2033–2044 doi:10.1016/j.neuroimage.2010.09.025. 20851191 10.1016/j.neuroimage.2010.09.025 PMC3065962

[28] Dom R, Malfroid M, Baro F (1976) Neuropathology of Huntington’s chorea: Studies of the ventrobasal complex of the thalamus. Neurology 26: 64–64 doi:10.1212/WNL.26.1.64. 10.1212/wnl.26.1.64 128708

[29] Thieben MJ, Diggins AJ, Good DC, Gomes L, Mahant N, et al (2002) The distribution of structural neuropathology in pre-clinical Huntington’s disease. Brain 125: 1815–1828 doi:10.1093/brain/awf179. 12135972 10.1093/brain/awf179

[30] Peng X, Wood CL, Blalock EM, Chen KC, Landfield PW, et al (2003) Statistical implications of pooling RNA samples for microarray experiments. BMC bioinformatics 4: 26 doi:10.1186/1471-2105-4-26. 12823867 10.1186/1471-2105-4-26 PMC166151

[31] Brown VM, Ossadtchi A, Khan AH, Gambhir SS, Cherry SR, et al (2002) Gene expression tomography. Physiol Genomicsgenomics 8: 159–167 doi:10.1152/physiolgenomics.00090.2001. 10.1152/physiolgenomics.00090.2001 11875194

[32] Ichikawa M, Okamura-Oho Y, Shimokawa K, Kondo S, Nakamura S, et al (2008) Expression analysis for inverted effects of serotonin transporter inactivation. BiochemBiophysResCommun 368: 43–49 doi:10.1016/j.bbrc.2008.01.041. 10.1016/j.bbrc.2008.01.041 18211820

[33] Edgar R (2002) Gene Expression Omnibus: NCBI gene expression and hybridization array data repository. Nucl Acids Res 30: 207–210 doi:10.1093/nar/30.1.207. 11752295 10.1093/nar/30.1.207 PMC99122

