# Supplementary figures and images for: Transcriptome Tomography for Brain Analysis in the Web-Accessible Anatomical Space
Source: PLoS One. 2012 Sep 19;7(9):e45373. doi: 10.1371/journal.pone.0045373 (PMC3446890; doi:10.1371/journal.pone.0045373)

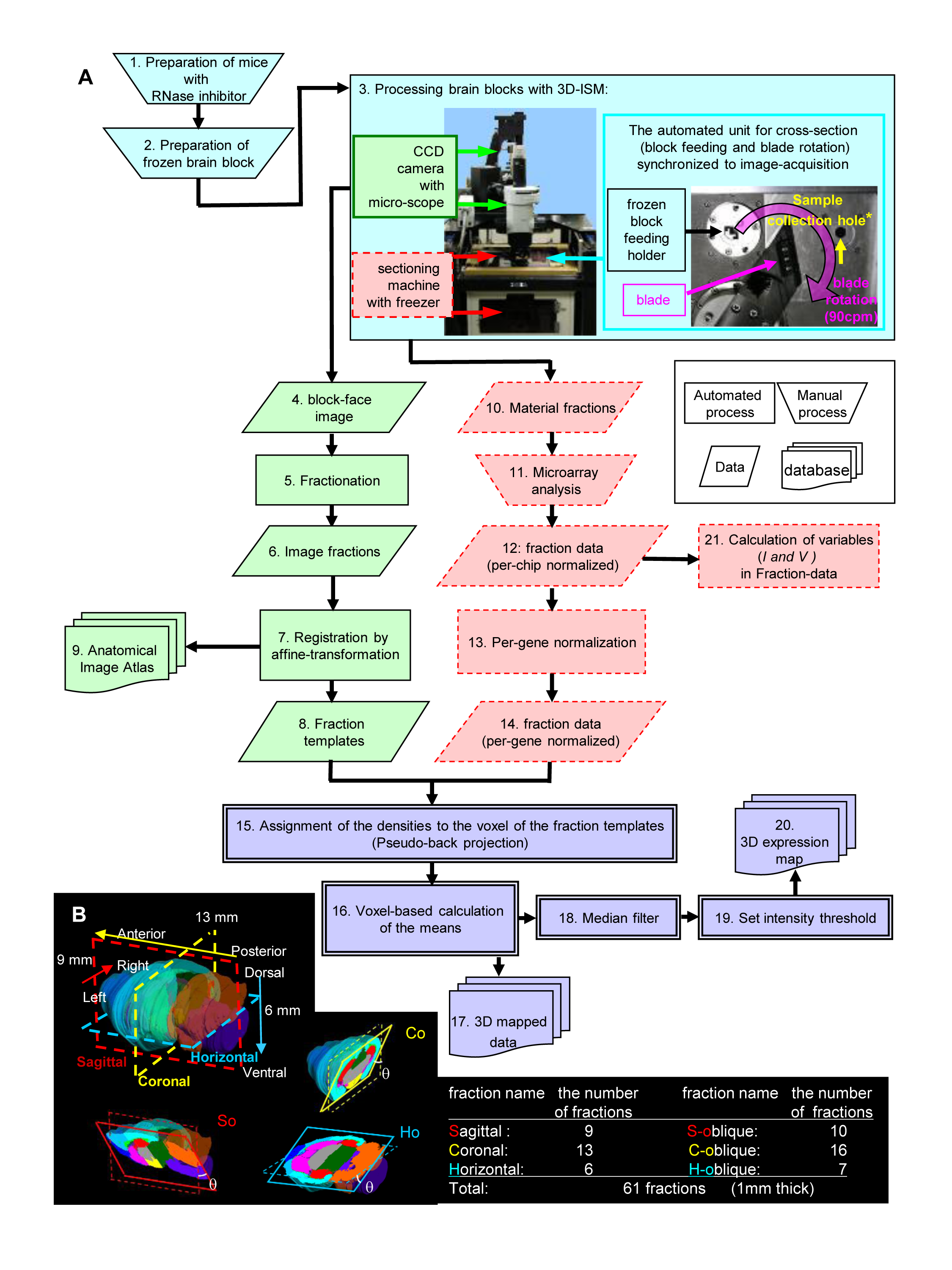

Supplement: Figure S1 — The framework for 3D mapping of transcriptome and analyses. (A) A flowchart of the processes for the framework. The steps of the processes are illustrated with shapes and colors. Legends for the shapes are in the right white panel and the colors are same as in Figure 1, with the light-blue color representing common processes. The processes are numbered and described in the Text S1. 3D-ISM was originally a device for sequential photography and was composed of three units: the image data acquisition device (indicated in green), the sectioning machine (in red) and the unit for synchronized sample feeding and blade rotation (in light-blue). A sample collection hole indicated by a yellow arrow is added to collect frozen sliced sections in batches (material fractions). (B) Body axes-based sections and the number of fractions. Arrows indicate the directions of the body axes-based sections of the model brain. The sectioning was performed in two groups of three series sectioned in each of orthogonal and slightly oblique to the orthogonal planes: S/C/H and So/Co/Ho, composed of 9/13/6 and 10/16/7 fractions, respectively, (61 fractions in total). (TIF) [file pone.0045373.s001.tif]
