# Supplementary material for: Transcriptome Tomography for Brain Analysis in the Web-Accessible Anatomical Space
Source: PLoS One. 2012 Sep 19;7(9):e45373. doi: 10.1371/journal.pone.0045373 (PMC3446890; doi:10.1371/journal.pone.0045373)
Supplement: Text S1 — Supporting methods for Transcriptome Tomography. (DOC) [file pone.0045373.s005.doc]

**Text S1. Supporting Methods for Transcriptome Tomography** (see a flowchart in Figure S1A).

**Brain sample preparation (Steps 1-4 and 10 in the flowchart)**

Brain samples were obtained from 8-week-old male C57BL/6J mice (Japan SLC) transcardially perfused with 7 ml of RNAlater (Ambion) in an anesthetized state. The samples were embedded in Tissue-Tek OCT compound (Sakura, Japan) containing 0.005 % toluidine blue and frozen at -80 °C. Using a sectioning machine, 3D-ISM: a block-face image was obtained before each 5 μm section was cut, then a serial set of sections (200 sections) was collected in batches as a fraction (5 μm x 200 = 1,000 μm in width) and a series of fractions was obtained using a whole brain (Video S1). The series of fractions (material fractions) were used for microarray analyses. This sectioning process was performed in six mice in each of six directions and the series of fractions were named after their sectioning planes as follows: Fractions C1-6; posterior to anterior, S1-13; left to right, H1-6; dorsal to ventral, Co1-7; slightly oblique to posterior/anterior, So1-16; slightly oblique to left/right and Ho1-7; slightly oblique to dorsal/ventral as shown in Figure S1B. In total, 61 fractions were obtained.

**Pseudo-tomography technique for generating 3D expression map (Steps 5-9, 13-20)**

The block-face images were used for the tomography technique in two ways. First, the images were fractionated into the 1-mm (5 μm x 200 surface images)-thick fractions (image fractions) to indicate the brain areas of the material fractions: the six anatomical images with fractions were applied to affine transformation to be located in the same x-y-z coordinates as each other (**image registration** to create fraction templates). Second, the transformed images of the horizontal (H) sectioning series at a 50 μm resolution were referred to as an Anatomical Image Atlas that showed the macroscopic anatomical information in the same coordinates as the fraction templates.

The fraction data produced as intensity values measured with microarray were per-gene normalized to the maximum and used for the tomography technique. A normalized intensity value for a gene-probe in a material fraction was assigned into (50μm)^3^ voxels of the corresponding image fraction in the fraction template (**pseudo-back projection**). The mean values of the six intensities in the six voxels of the fraction templates, which have the same x-y-z coordinates, were calculated and linearly converted to 8-bit color intensity grades (**tomographic reconstruction** to create **3D mapped data**). After smoothing by a 7x7x7 median filter twice, a threshold was defined at 80 % of the maximal intensity grade value (80 % cutoff filter) and areas above the threshold were indicated with 27 color grades to eliminate intensities of false positives caused by the tomography techniques (**3D expression map**). 3D reconstruction was performed using an image processing software, VCAT (downloadable from <http://vibrism.riken.jp/index2.php>).

**Microarray procedures and Fraction data analyses (Steps 11-12 and 21, respectively)**

Microarray experiments using standard protocol were described in the Materials and Methods section. Calculation of Sets of variables using log-transformed fraction data was also described in the section.
