# Supplementary material for: Transcriptome Tomography for Brain Analysis in the Web-Accessible Anatomical Space
Source: PLoS One. 2012 Sep 19;7(9):e45373. doi: 10.1371/journal.pone.0045373 (PMC3446890; doi:10.1371/journal.pone.0045373)
Supplement: Text S2 — Supporting methods for accuracy estimation of the present tomography technique with a computational experiment using test spheres. (DOC) [file pone.0045373.s006.doc]

**Text S2. Supporting Methods for Accuracy Estimation of the Present Tomography Technique with a Computational Experiment** **Using Test Spheres** (see Figure 2).

The test sphere was a phantom of gene expression randomly selected in the ViBrism space, and the diameter was 20 voxels (1,000 μm). Assuming that the volume of the each test sphere, which is equal to the total amount of a gene, was 1.0, the volume of the test sphere portion located in the fraction of the six fraction templates were calculated. Then, the volume was evenly divided and allocated to the voxels of the fraction. The allocated volume was equivalent to the expression intensity values assigned in the voxels of the fraction template. The following calculations were the same as those for the first dataset, and the reconstructed results were visualized by volume rendering in a gray scale with or without an 80 % cutoff filter. Areas with voxel intensities above the cutoff filter were denoted as reconstructed areas.
